# The Safety and Efficacy of the Modified Single Incision Non-thoracoscopic Nuss Procedure for Children With Pectus Excavatum

**DOI:** 10.3389/fped.2022.831617

**Published:** 2022-02-08

**Authors:** Jishuo Song, Quan Wang, Zhengxia Pan, Chun Wu, Yonggang Li, Gang Wang, Jiangtao Dai, Linyun Xi, Hongbo Li

**Affiliations:** ^1^Department of Day Surgery Children's Hospital of Chongqing Medical University, National Clinical Research Center for Child Health and Disorders, Ministry of Education Key Laboratory of Child Development and Disorders, Chongqing Key Laboratory of Pediatrics, Chongqing, China; ^2^Department of Cardiothoracic Surgery Children's Hospital of Chongqing Medical University, National Clinical Research Center for Child Health and Disorders, Ministry of Education Key Laboratory of Child Development and Disorders, Chongqing Key Laboratory of Pediatrics, Chongqing, China

**Keywords:** pectus excavatum (PE), Nuss procedure, single incision, non-thoracoscopic, children

## Abstract

**Background:**

This study described and evaluated the safety and efficacy of a modified single incision non-thoracoscopic Nuss procedure in pectus excavatum (PE) children.

**Methods:**

PE patients undergoing the non-thoracoscopic Nuss procedure at the Children's Hospital of Chongqing Medical University between January 2017 and December 2020 were retrospectively enrolled. The patients were divided into two groups according to operation procedures: the double incision Nuss (DN) group and the modified single incision Nuss (SN) group. Propensity score matching (PSM) was applied before evaluation of operative and postoperative characteristics to reduce selection bias.

**Results:**

Of the 502 patients included, 261 were enrolled in the DN group, and 241 in the SN group. The operation time [35.0 (30.0–40.0) vs. 50.0 (40.0–55.0) minutes, *P* < 0.001] and postoperative inpatient stay [7.0 (6.0–8.0) vs. 7.0 (7.0–8.0) days, *P* < 0.001] of the patients in the SN group after PSM were significantly shorter than those of the patients in the DN group after PSM. Moreover, median blood loss was significantly less in the SN group after PSM than that in the DN group after PSM [2.0 (1.0–5.0) vs. 5.0 (2.0–5.0) ml, *P* < 0.001]. There were no significant differences in the incidence of complications between the two groups (*P* > 0.05). Bar removal was performed in 85 patients in the SN group within 24–42 months after surgery. Additionally, the SN group patients had a significantly lower Haller index (HI) after bar removal [2.36 (2.15–2.55) vs. 3.76 (3.18–4.26), *P* < 0.001] compared to the initial HI.

**Conclusions:**

The modified procedure is safe and effective for children with PE and is worthy of clinical application.

## Introduction

Pectus excavatum (PE) is a common thoracic deformity, with an incidence of ~0.1–0.8% and a male to female ratio of 5:1 (1, 2). Most infants and young children with PE are asymptomatic, but as they get older and become more active, PE may lead to lack of endurance, exercise intolerance, shortness of breath during exertion, and abnormal psychological function, even results in psychosocial disorders (1, 3). Moreover, severe PE may impair cardiopulmonary function and affect the growth of children (2, 4, 5). Spontaneous resolution of PE is unlikely, and a more typical process is the worsening of chest wall depression during rapid vertical growth and puberty (6). Therefore, surgical approaches are the most effective methods for correcting PE. The invasive Ravitch procedure and the minimally invasive Nuss procedure are the described surgical treatments for PE (7, 8).

The Nuss procedure for the minimally invasive repair of PE was first reported in 1998 (8). Compared with the Ravitch procedure, this approach has the advantages of less trauma, better cosmetic effects, fewer serious complications, and excellent surgical effects; therefore, the Nuss procedure has replaced open surgery and become the main surgical procedure for treating PE (8, 9). Although the Nuss procedure has several advantages, some studies have revealed that it had complications of bar migration, pneumothorax, and hemothorax and lead to a significant decrease in postoperative pulmonary volume and function (10, 11). Thus, several studies have explored modified techniques for the Nuss procedure in patients. A systematic review presented recent modifications of the Nuss procedure which aimed to pursue surgical safety and minimize complications (12). Two studies described a modified Nuss procedure that was performed through a single lateral thoracic incision to acquire aesthetic outcomes (13, 14). However, these single incision procedures were performed with thoracoscopic guidance, and few studies have explored the efficacy and safety of single incision non-thoracoscopic Nuss procedure in children. Thus, we conducted this retrospective study to investigate the advantages, safety and efficacy of the modified single incision non-thoracoscopic Nuss procedure in PE children.

## Materials and Methods

### Study Population and Clinical Information

We performed a retrospective, observational cohort study at the Children's Hospital of Chongqing Medical University, a 2,480-bed [95 cardiothoracic surgery beds] tertiary teaching hospital in Chongqing, China, ranked among the top three domestic children's hospitals (rank list: http://top100.imicams.ac.cn/home). A total of 502 patients hospitalized in cardiothoracic surgery between January 2017 and December 2020 were retrospectively included in the study. The PE patients enrolled in this study met the following inclusion criteria: (I) aged 3–18 years (3, 15, 16), (II) patients with PE who underwent the Nuss surgery. The exclusion criteria included any of the following: (I) patients who underwent surgery with thoracoscopic guidance, (II) treatment with a single bar was ineffective, (III) patients receiving a thoracic surgery previously who required an additional subxiphoid incision attribute to difficult dissection of retrosternal adhesions, (IV) patients with severe asymmetric chest and complex chest wall anomalies, (V) patients received other operations concurrently, (VI) patients with incomplete clinical information. The recurrent PE is a non-exclusion criterion for this modified procedure. Trained staff extracted patient data from electronic medical databases. Demographic data, Haller index (HI) values, recurrent PE status, ultrasonic cardiogram (UCG), electrocardiogram (ECG), pulmonary function tests, surgery characteristics (duration of surgery, estimated blood loss), postoperative complications, duration of postoperative stay, follow-up time, and bar removal time were retrospectively collected.

### Classifications and Definitions

According to the differences in incisions, patients were divided into two subgroups: (I) a double incision Nuss (DN) group and (II) a modified single incision Nuss (SN) group. PE was defined as an anatomic depression of the chest wall and it was diagnosed based on a history of concave deformity of the anterior thorax and physical examination (2). The severity of the chest wall deformity was assessed by the HI values which were calculated using computed tomography (CT) or/and chest radiograph (17). The indications for surgical treatment included the following criteria (18): (I) the HI > 3.25, (II) shortness of breath or exercise intolerance, (III) mitral valve prolapse, bundle branch block or other cardiac pathology attribute to PE, (IV) abnormal pulmonary function tests, (V) substantial psychosocial concerns, (VI) cardiac or pulmonary compression on CT or UCG, (VII) progression of deformity, (VIII) previous repair failure. Of these criteria, the 1st to the 5th criteria are the main criteria and the last three are the minor criteria (19). In this study, PE patients undergoing surgery met at least two criteria, of which it must meet at least one of the major criteria.

### Operative Methods

**Figure 1:**
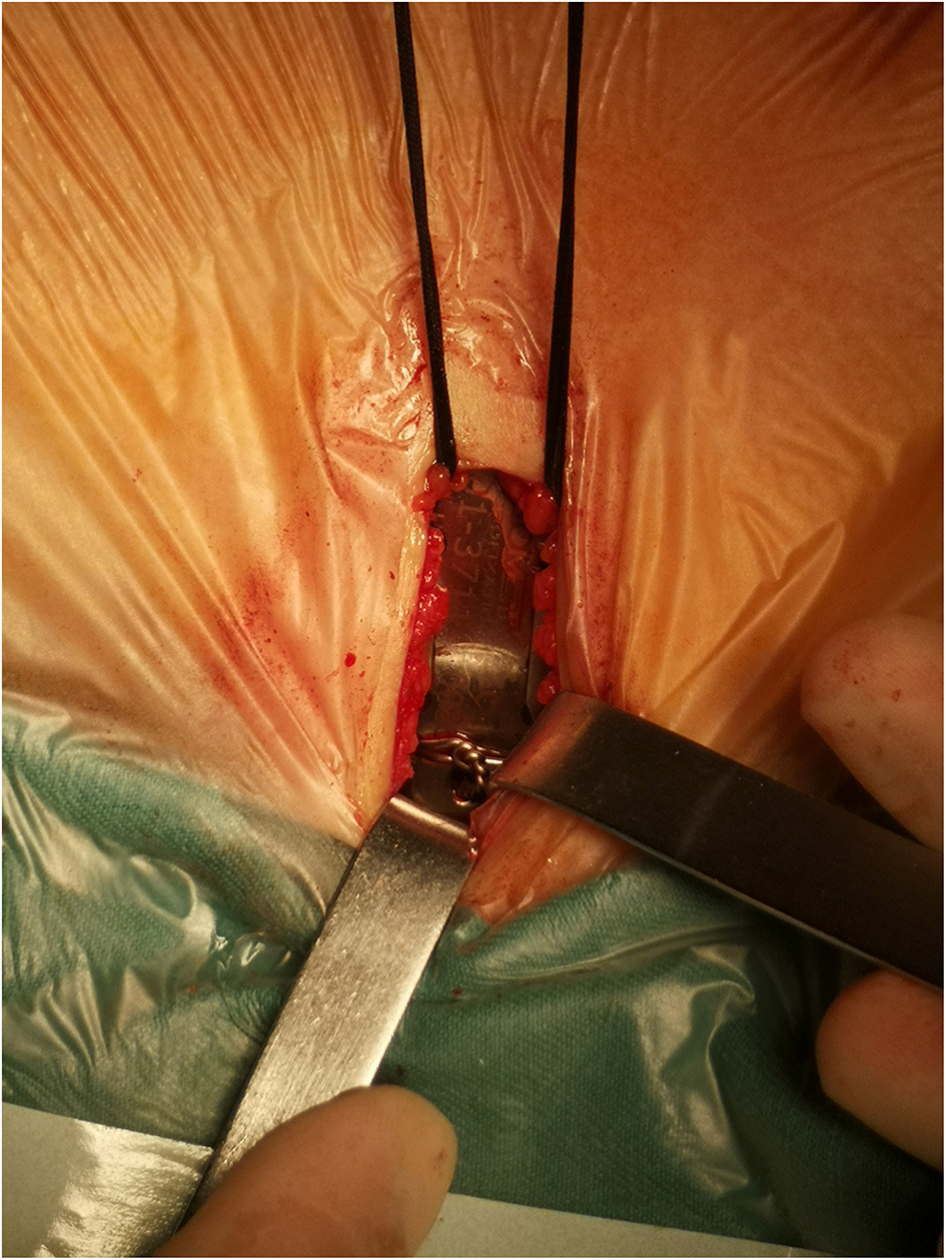
The stabilizer was fixed with a steel wire encircled as “8”-shaped on the groove of the bar.

All patients were treated with pectus support bar system (Biomet Microfixation Inc., Jacksonville, FL, USA) including orthopedic bars and bar stabilizers, and instruments including bar introducers, bar flippers and shaping forceps. The selected size bar was precurved to fit the morphology of the chest. All patients underwent general anesthesia with tracheal intubation and were placed in the supine position with an arm abduction of 90° prior to the start of the surgery. A single transverse incision 2–3 cm was made in the left midaxillary line at the lowest point of thoracic depression. And the subcutaneous tissue was separated to reach the costal membrane. After that, blunt dissection of tissue from the upper surface of the ribs to the lower pectoralis layer to establish a transverse tunnel from the incision forward to the hinge point (Supplementary Video 1). Then, the bar introducer was used to dissect and establish the subcutaneous tunnel on the right side of the chest. It was important that the right chest tunnel was generous enough to allow the bar traverse and rotation (Supplementary Video 2). The bent bar was then passed along the tunnel with the convexity of the bar facing posterior. Once in position, the bar was rotated 180° with the bar flipper so that the convexity could face anterior and elevate the sternum (Supplementary Video 3). The bar stabilizer was set at the left end of the bar, and it was fixed with a steel wire encircled as “8”-shaped on the groove of the bar (Figure 1, Supplementary Video 4). In addition, we reinforced the stabilizer to a rib with zero polydioxanone (PDS) sutures (Supplementary Video 5). The incision was closed by layers. It was unnecessary to place any thoracic drainage.

### Postoperative Management

**Figure 2:**
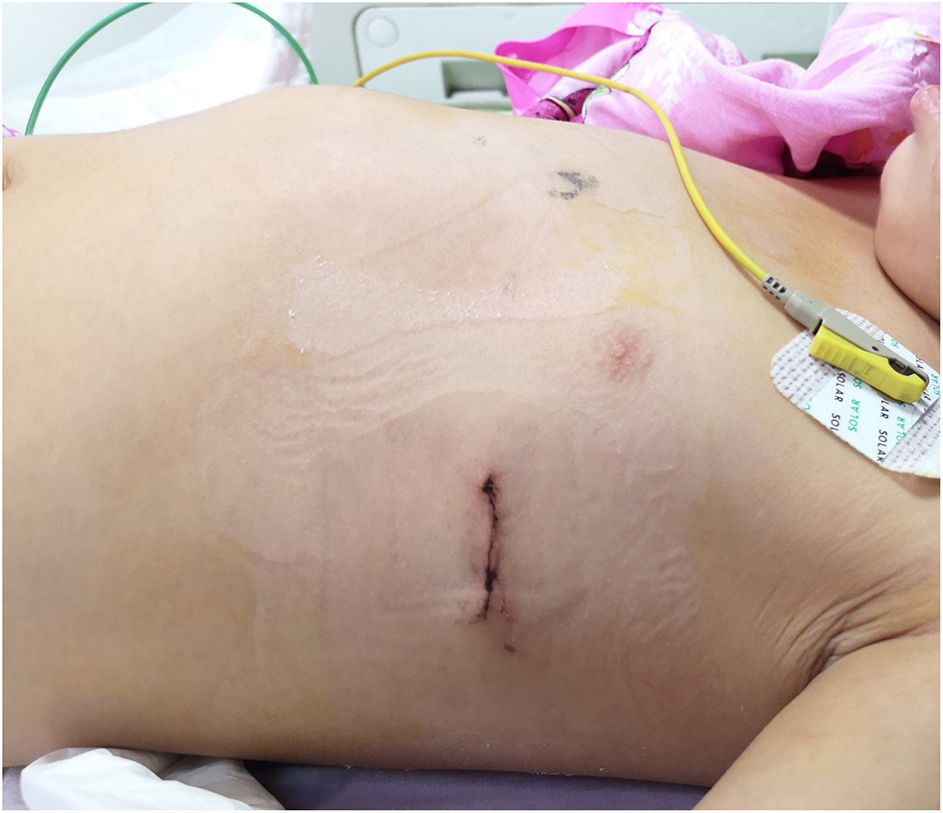
The appearance of the left chest wall incision and the corrected chest 1 day after surgery.

**Figure 3:**
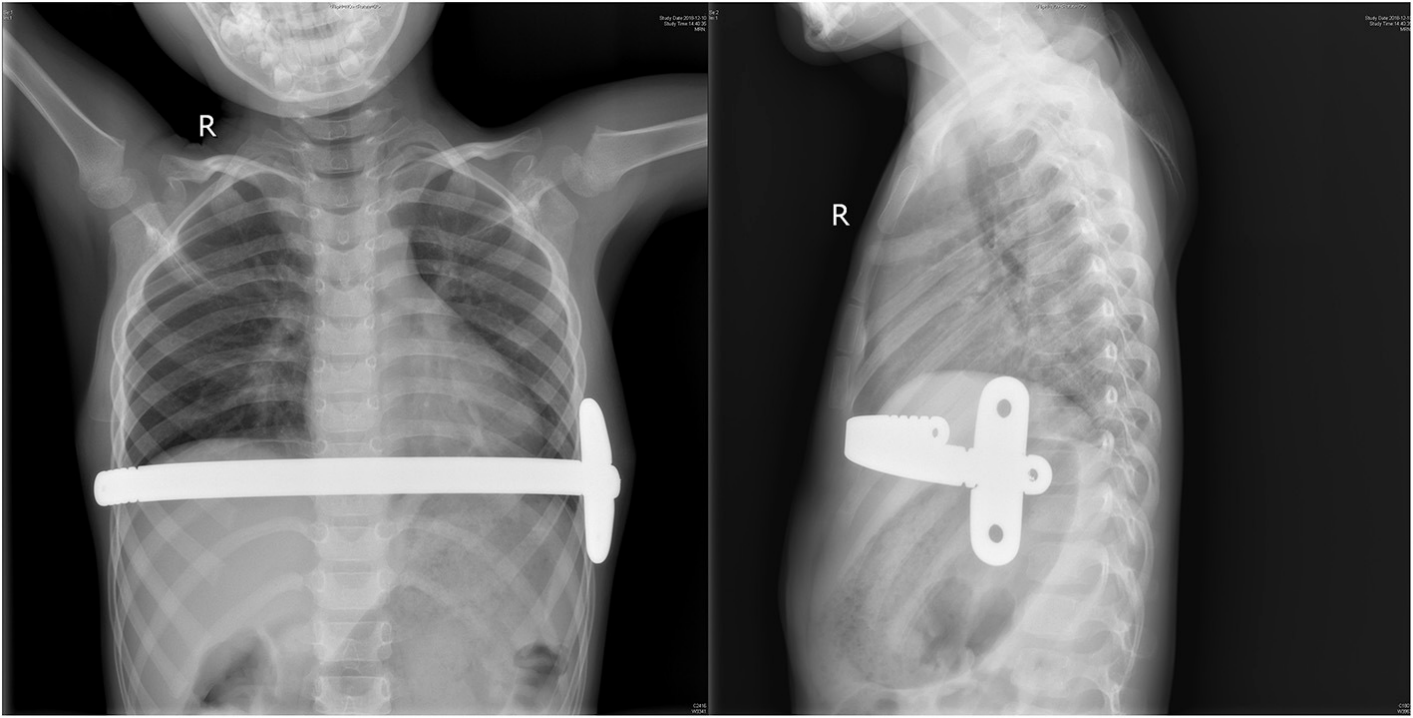
The chest radiograph showed that bar placement and fixation 3 days after surgery.

All patients were monitored in the cardiac intensive care unit (CICU) at least for 1 day after surgery (Figure 2). All patients received antibiotics and analgesics. The patients were required to lie supine to avoid putting pressure on their chests. Early ambulation was encouraged, and the patients should keep their heads and chests up to avoid bending the lumbar spine. Chest radiography was conducted on every patient to confirm the location of the bar and evaluate the surgical effect (Figure 3).

### Follow-Up

All patients were told to undergo regular postoperative follow-up in the Outpatient Department during the 1st, 3rd, and 6th months and during the 1st and 2nd years. In our center, we decided the time span for bar removal based on the results of previous studies (15, 16, 20, 21), the patients' condition and our experience. The bar was removed through the previous incision 2–3.5 years postoperatively. After bar removal, follow-up was performed during the 1st and 6th months and during the 1st and 2nd years. During each visit, the surgeon performed a careful physical examination; chest radiography was also conducted routinely to assess the chest appearance. Once the bar was removed, the CT or chest radiography was conducted to calculate the postoperative HI.

### Statistical Analysis

Continuous variables were expressed as the median with interquartile ranges (IQRs), and categorical variables were expressed as the counts (*n*) and percentages (%). The Mann–Whitney *U*-test was used to compare the continuous variables between two groups. Pearson's chi-square or Fisher's exact tests were used to compare the categorical variables. Propensity score matching (PSM) was performed to reduce the potential for confounding by baseline factors between the two groups. A multivariate logistic regression model, including age, sex, weight, HI, UCG, ECG, pulmonary function tests, and recurrent PE, was used to calculate the propensity scores. The 1:1 nearest neighbor matching method was employed, and the caliper was set as 0.2. PSM was carried out without replacement. Non-matched patients were discarded. The propensity score distribution was evaluated to detect sufficient overlap between two groups to ensure comparability. HI values before treatment and after bar removal were compared by paired *t*-tests. A *P* < 0.05 was regarded as statistically significant. IBM SPSS Statistics 22.0 (IBM Corp., Armonk, NY, USA) was used for all statistical analyses.

## Results

### Patient Demographics and Preoperative Characteristics

During a period of 4 years, a total of 502 patients with PE who underwent the non-thoracoscopic Nuss procedure were included: 241 patients underwent the modified single incision non-thoracoscopic procedure, and 261 underwent the double incision non-thoracoscopic procedure. Twenty-one of all patients underwent PE surgery and experienced recurrence: 19 patients underwent the Ravitch procedure, and two patients underwent the Nuss procedure at the first treatment. Forty-six patients showed cardiac compression, cardiac enlargement or decreased left ventricular diastolic function on UCG. A total of 148 patients had abnormal ECG results, including complete or incomplete right bundle branch block, T-wave change, atrioventricular block, and right or left deviation of the electrical axis. And 162 patients showed abnormal pulmonary function tests, including restrictive ventilation disorders, obstructive ventilation disorders, and mixed ventilation disorders.

### Operative and Postoperative Characteristics

**Figure 4:**
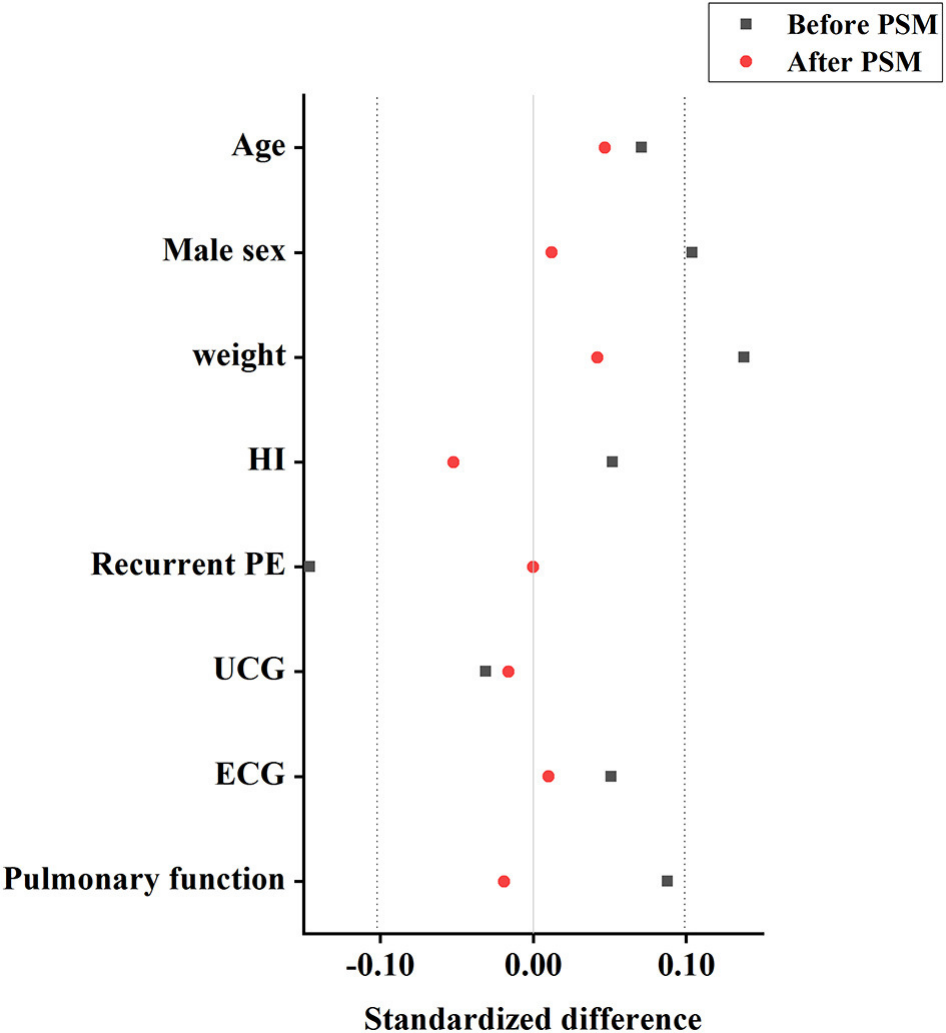
Improvement in covariate balance by propensity scoring. Plot showing improvement in standardized differences in means for all measured covariates in patients in the propensity-matched cohorts. An absolute standardized difference <0.10 shows adequate matching. HI, Haller index; PE, pectus excavatum; UCG, ultrasonic cardiogram; ECG, electrocardiogram; PSM: Propensity score matchaing.

All 502 patients who underwent the Nuss procedure did not occur intraoperative life-threatening events, such as cardiopulmonary injury, cardiac arrest, and fatal bleeding. PSM was performed to minimize the potential for confounding by baseline factors between two groups. A total of 220 matched patients included in the following analysis. Two group patients had well-matched baseline characteristics (Table 1), and the absolute standardized differences in the means were <0.10 (Figure 4). Operative and postoperative characteristics after PSM are presented in Table 2. The median operation time and postoperative inpatient stay in the SN group was significantly shorter than those in the DN group [35.0 (30.0–40.0) vs. 50.0 (40.0–55.0) minutes, *P* < 0.001; 7.0 (6.0–8.0) vs. 7.0 (7.0–8.0) days, *P* < 0.001, respectively]. Moreover, the SN group patients had significantly less blood loss when compared to the DN group [2.0 (1.0–5.0) vs. 5.0 (2.0–5.0) ml, P <0.001]. There were no remarkable differences in postoperative complications (pneumonia, asymptomatic pneumothorax, pleural effusion, and incision infection) between two groups after PSM (*P* > 0.05; Table 2).

**Table 1:** Demographics and clinical characteristics.

**Variables**	**Before PSM**			**After PSM**		
	**SN (*n* = 241)**	**DN (*n* = 261)**	**Standardized differences**	**SN (*n* = 220)**	**DN (*n* = 220)**	**Standardized differences**
Age (years), median (IQR)	8.17 (5.08–13.13)	7.33 (5.08–12.71)	0.071	7.63 (5.00–12.90)	7.04 (5.00–12.58)	0.047
Male sex, n (%)	198 (82.2)	204 (78.2)	0.104	178 (80.9)	177 (80.5)	0.012
Weight (kg), median (IQR)	23.50 (18.00–41.00)	22.00 (18.00–35.00)	0.138	22.00 (18.00–40.00)	22.00 (18.00–35.00)	0.042
HI, median (IQR)	3.68 (3.17–4.22)	3.47 (3.01–4.09)	0.052	3.65 (3.16–4.18)	3.49 (3.01–4.10)	−0.052
Recurrent PE, n (%)	7 (2.9)	14 (5.4)	−0.146	5 (2.3)	5 (2.3)	0
UCG, *n* (%)	21 (8.7)	25 (9.6)	−0.031	19 (8.6)	20 (9.1)	−0.016
ECG, *n* (%)	74 (30.7)	74 (28.4)	0.051	66 (30.0)	65 (29.5)	0.010
Pulmonary function, *n* (%)	83 (34.4)	79 (30.3)	0.088	68 (30.9)	70 (31.8)	−0.019

*DN, double incision Nuss; SN, modified single incision Nuss; PSM, propensity score matching; IQR, interquartile range; HI, Haller index; PE, pectus excavatum; UCG, ultrosonic cardiogram; ECG, electrocardiogram*.

**Table 2:** Operative and postoperative characteristics of SN and DN groups after PSM.

**Variables**	**SN (*n* = 220)**	**DN (*n* = 220)**	***P***
Duration of surgery (minutes), median (IQR)	35.0 (30.0–40.0)	50.0 (40.0–55.0)	<0.001
Blood loss (ml), median (IQR)	2.0 (1.0–5.0)	5.0 (2.0–5.0)	<0.001
Duration of postoperative stay (days), median (IQR)	7.0 (6.0–8.0)	7.0 (7.0–8.0)	<0.001
**Postoperative complications**, ***n*** **(%)**			
Pneumothorax	1 (0.5)	2 (0.9)	1.000
Pleural effusion	4 (1.8)	2 (0.9)	0.685
Pneumonia	16 (7.3)	26 (11.8)	0.105
Incision infection	3 (1.4)	9 (4.1)	0.079
Bar displacement	2 (0.9)	2 (0.9)	1.000

*DN, double incision Nuss; SN, modified single incision Nuss; PSM, propensity score matching; IQR, interquartile range*.

### Follow-Up and Clinical Outcomes

The follow-up period was 10–58 months. During the follow-up period, bar displacement that required reoperation occurred in five patients, two of whom were in the SN group. However, there was no remarkable differences in the incidence of bar displacement between two groups after PSM (*P* = 1.000; Table 2). Complications, such as acquired scoliosis, metal allergy, and deformity recurrence after bar removal, did not occur. All patients had excellent chest appearances after surgery. The bar was removed in 85 patients in the SN group and 110 patients in the DN group within 24–42 months after surgery. In our study, 185 patients' time span for bar removal was 2–3 years, 10 patients delayed the time due to academic or respiratory infections. The follow-up period after bar removal was 3–20 months. Table 3 showed the median HI 3 months after bar removal had a significantly decrease when compared to the HI before operative correction both in the SN group and DN group [2.36 (2.15–2.55) vs. 3.76 (3.18–4.26), *P* < 0.001; 2.37 (2.05–2.65) vs. 3.43 (3.03–4.12), *P* < 0.001, respectively]. During the follow-up period, 195 patients with bar removal obtained an excellent appearance in two groups.

**Table 3:** HI pre-treatment and HI after bar removal in SN and DN groups.

**Variables**	**SN (*n* = 85)**	**DN (*n* = 110)**
HI pre-treatment, median (IQR)	3.76 (3.18–4.26)	3.43 (3.03–4.12)
HI after bar removal, median (IQR)	2.36 (2.15–2.55)	2.37 (2.05–2.65)
*T*	13.066	14.425
*P*	<0.001	<0.001

*HI, Haller index; SN, modified single incision Nuss; DN, double incision Nuss; IQR, interquartile range*.

## Discussion

Although the Nuss procedure has several advantages, some studies have revealed that it has some shortcomings, such as intraoperative cardiac injury, complications of bar displacement, pneumothorax and prolonged postoperative pain (16, 22). Thus, various modified methods and techniques have been used in the Nuss procedure over the past 20 years. Many improvements have focused on how to lift the depressed sternum more precisely and insert the bar in a safer manner, such as assistance with a bilateral thoracoscopy, adding a subxiphoid incision, and sternal elevation by using a vacuum bell or crane techniques (20, 23, 24). The application of stabilizers and modified fixation techniques have effectively decreased the postoperative bar displacement (15, 25). However, the improvement in cosmetic effects were relatively limited. A survey from the Chinese Association of Thoracic Surgeons revealed that the most common reason for patients to require surgery was psychological discomfort due to PE deformity, and cosmetic requests came second (26). The minimally invasive procedure has a better cosmetic result. Furthermore, the minimally invasive procedure can promote self-esteem and improve psychosocial functions.

To the best of our knowledge, this study is one of the largest studies to investigate the advantages, safety and efficacy of the modified single incision non-thoracoscopic Nuss procedure. In our study, the SN group patients receiving bar removal obtained an excellent chest appearance without recurrence. In addition, the SN group patients had an advantage of fewer incisions compared to the DN group patients, which might effectively decrease surgery time and diminish intraoperative trauma. Our study demonstrated that the postoperative hospital stay in the SN group was significantly shorter than that in the DN group. A possible explanation for shorter postoperative hospital stay was that the SN group patients might experience less pain when compared to the DN group. A previous study showed that the postoperative pain was associated with breathing exercises and other activities that promoted postoperative recovery (6). In addition, a systematic review demonstrated that a longer operative duration was associated with increased wound contamination and might lead to a higher infection rate and longer healing time (27). Although no significant differences in the incision infection rates were found between SN and DN groups (*P* = 0.079). The results showed that the incision infection rate of the SN group was 1.4%, which was lower than that in the DN group (4.1%). Thus, our study showed that a single incision and less intraoperative trauma could relieve postoperative incision pain, decrease the incision infection rates and shorten the postoperative hospital time.

Some studies have demonstrated that there was a risk of lung or cardiac injury when introducers and orthopedic bars pass back and forth, so some thoracic surgeons insisted that it was necessary to perform operations with the guidance of thoracoscope in order to ensure safety and reduce complications (28, 29). Previous studies have reported the modification of using a single lateral incision with thoracoscopic guidance (13, 14). However, the Nuss surgery was initially performed without thoracoscopic guidance (8). Moreover, studies by Han et al. (30) and Sacco Casamassima et al. (31) have shown that compared with thoracoscopic-assisted procedures, non-thoracoscopic procedure could simplify the operation and save surgery time in both children and adults. We have completed more than 1,000 cases of non-thoracoscopic Nuss procedures over the past decade in our center and no intraoperative life-threatening events occurred (3). We chose the left lateral chest incision rather than the right side in order to avoid cardiac injury in the modified procedure.

When the introducer passed through the intercostal space and enters the retrosternal space from the left approach, the introducer had crossed most of the region corresponding to the heart. Thus, we think the risks of cardiac or pericardial injury could be reduced with the left lateral approach. The end of the introducer and bar should be adjusted to make the concavity upwards when entering the retrosternal space, and the introducer and bar should be moved closely to the back of the sternum in a light and gentle manner during operation (Supplementary Video 2). In the movement process of the introducer and bar, the ECG should be observed carefully. A survey from the Chinese Association of Thoracic Surgeons indicated that about half of the responders undertook cardiac injury prevention strategies, and only 49.41% of them adopted thoracoscopy (26). In addition, a previous study has pointed out that thoracoscopy alone did not eliminate the risk of cardiac puncture (32). In our study, no intraoperative life-threatening events occurred in patients receiving the modified single incision non-thoracoscopic Nuss procedure. Hence, our modified procedure without thoracoscopic was safe.

The accurate selection and evaluation of patients is important to reduce the intraoperative risks. In terms of the surgeons' choice, the modified procedure was completed by a left single incision without thoracoscopic guidance, so the operation might require proficient and experienced surgeons. For beginners, simple and symmetrical patients should be selected for this modified procedure with the guidance of an experienced surgeon. In the selection of patient age, we chose children aged 3–18 for surgery. We had successfully performed modified procedures in these children. The study of Park et al. (15, 16) has demonstrated that early repair of PE in patients ≥3 years old was safe and effective, and early repair of PE was associated with avoiding asymmetry transformation of the deformity and improving the patients' growth potential. Furthermore, our previous study have indicated that age was a risk factor for the occurrence of psychosocial disorders, and patients with severe PE for a longer time had more serious psychosocial disorders and a hard time recovering, and most patients did not perceive their deformity before the age of 4 years (3). Therefore, it is reasonable that children with PE receive Nuss procedure at the age of 3–18 years. In addition, the exclusion criteria are undoubtedly very important. In clinical practice, some patients might not be suitable for the modified procedure. Patients who meet the following three conditions are not candidates for this modified surgery. Firstly, PE patients with severe asymmetric chest and complex chest wall deformity should not be considered for the modified procedure due to the complex operation. Secondly, PE patients with chest deformity over multiple rib levels or stiff chest wall cannot benefit much from the single bar. Thirdly, some PE patients undergoing thoracic surgery previously are not suitable for this modified surgery because these patients might require an additional subxiphoid incision due to difficult dissection of retrosternal adhesions.

Whether it is a standard Nuss procedure or any variety of modified Nuss procedures, bar fixation is the key factor in ensuring the surgical effect. As our procedure adopted a single incision and no bar stabilizer was placed at the contralateral side, some surgeons raised concerns that the use of a unilateral bar stabilizer might increase bar displacement and recurrence. However, only 2 patients in the SN group suffered from bar displacement in the follow-up, which indicated that the use of a unilateral stabilizer was effective. Similar to our findings, many reports have shown that a unilateral bar stabilizer provided enough support to ensure the stability of the bar, and some thoracic surgeons believed that the stability of the bar depended not only on the stabilizer but also on the fulcrum effect provided by the bar spanning 2–3 ribs on the bilateral sides (13, 16). Thus, in order to reach the fulcrum effect, a suitable subcutaneous tunnel and appropriate location of the bar is necessary. Furthermore, compared with bilateral stabilizer fixation, single stabilizer fixation could reduce consumables and expenses, and removal of the bar can be completed by a single incision.

The introduction of pericostal sutures combined with the bar stabilizer reduced the bar displacement rates (20). On the basis of the bar stabilizer, we encircled the groove of the bar with steel wire to prevent the stabilizer from sliding outward, which was the most important improvement measure to prevent bar displacement (Figure 1). At the same time, zero PDS sutures were placed around the stabilizer and the underlying rib to ensure the stability of the bar and the stabilizer, which prevented the bar from rotating. Kelly et al. (12) showed that the bar displacement requiring reoperations was 1.8%. However, the bar displacement rate in our study was <1%, which indicted the modified procedure was safe and effective.

There are several limitations to this study. Firstly, this was a retrospective single-institution study, and it was difficult to control all the variables to avoid biases. Secondly, the follow-up period was limited, and the sample size was relatively small, especially for patients who completed the modified Nuss procedure and bar removal. Thirdly, the modified procedure requires surgeons to be highly proficient and experienced. A larger multicenter prospective study is needed to explore the safety and efficacy of the modified single incision non-thoracoscopic Nuss procedure.

## Conclusions

The modified procedure is safe and effective for children with PE and is worthy of clinical application.

## Data Availability Statement

The raw data supporting the conclusions of this article will be made available by the authors, without undue reservation.

## Ethics Statement

The studies involving human participants were reviewed and approved by the Institutional Review Board of Children's Hospital of Chongqing Medical University. Written informed consent to participate in this study was provided by the participants' legal guardian/next of kin. Written informed consent was obtained from the individual(s), and minor(s)' legal guardian/next of kin, for the publication of any potentially identifiable images or data included in this article.

## Author Contributions

JS drafted and revised the manuscript, carried out clinical data collection, data analysis and interpretation, and contributed to the design of the study. QW critically revised the manuscript and contributed to the design of the study. ZP, CW, YL, GW, and JD critically revised the manuscript. LX contributed to the design of the study. HL conceptualized and designed the study, critically revised the manuscript, and oversaw the creation of the final manuscript. All authors approved the final manuscript as submitted and agree to be accountable for all aspects of the work.

## Funding

This study was supported by the National Clinical Research Center for Child Health and Disorders funding project (NCRCCHD-2021-YP-17).

## Conflict of Interest

The authors declare that the research was conducted in the absence of any commercial or financial relationships that could be construed as a potential conflict of interest.

## Publisher's Note

All claims expressed in this article are solely those of the authors and do not necessarily represent those of their affiliated organizations, or those of the publisher, the editors and the reviewers. Any product that may be evaluated in this article, or claim that may be made by its manufacturer, is not guaranteed or endorsed by the publisher.

## Abbreviations

PE: pectus excavatum
CT: computed tomography
UCG: ultrasonic cardiogram
ECG: electrocardiogram
HI: Haller index
PDS: polydioxanone
CICU: cardiac intensive care unit
DN: double incision Nuss
SN: modified single incision Nuss
IQR: interquartile range
PSM: propensity score matching.
